# Drift–diffusion modeling reveals that masked faces are preconceived as unfriendly

**DOI:** 10.1038/s41598-023-44162-y

**Published:** 2023-10-09

**Authors:** Martijn J. Mulder, Franziska Prummer, David Terburg, J. Leon Kenemans

**Affiliations:** 1 Department of Experimental Psychology, Helmholtz Institute, Utrecht University, Utrecht, The Netherlands; 2 School of Computing and Communications, Lancaster University, Lancaster, UK

**Keywords:** Psychology, Human behaviour

## Abstract

During the COVID-19 pandemic, the use of face masks has become a daily routine. Studies have shown that face masks increase the ambiguity of facial expressions which not only affects (the development of) emotion recognition, but also interferes with social interaction and judgement. To disambiguate facial expressions, we rely on *perceptual* (stimulus-driven) as well as *preconceptual* (top-down) processes. However, it is unknown which of these two mechanisms accounts for the misinterpretation of masked expressions. To investigate this, we asked participants (*N* = 136) to decide whether ambiguous (morphed) facial expressions, with or without a mask, were perceived as friendly or unfriendly. To test for the independent effects of *perceptual* and preconceptual biases we fitted a drift–diffusion model (DDM) to the behavioral data of each participant. Results show that face masks induce a clear loss of information leading to a slight *perceptual* bias towards friendly choices, but also a clear preconceptual bias towards unfriendly choices for masked faces. These results suggest that, although face masks can increase the perceptual friendliness of faces, people have the prior preconception to interpret masked faces as unfriendly.

## Introduction

During the COVID-19 Pandemic, wearing a face mask has become part of our daily life as it restricts the spread of the SARS-CoV-2 virus^1,2^. Since facial expressions play an important role in our social communication^3–6^, wearing such a mask might also affect social conduct. For example, when we accidently step on someone’s toes in the supermarket, apologizing with a friendly smile might not be sufficient to save the situation. Indeed, recent studies show that facial masks affect face perception, recognition and identification^7–14,14–16^, interfere with social interaction and social judgments^7,13,14^ and might even hamper the development of emotion recognition in children^12,17^.

Importantly, face masks not only reduce the amount of sensory information, but potentially also influence the classification of facial expression in a more systematically biased way by adding perceptual information (e.g., masks could make people look more angry or sad), and/or evoking preconceptions about obscured emotional expressions. In other words, the detrimental effects face masks have on our social interactions^7,13,14^ can stem from perceptual aspects of emotion recognition, and potentially from pre-existing biases driven by previously held negative connotations associated with facial masks. Given the general social importance of facial emotion recognition it is vital to disentangle such perceptual from preconceptional biases. Especially since face masks have become part of our daily routine, awareness of how this can impact our social communication might help to counter the potential negative consequences.

Evidence showing misinterpretation of emotional expressions due to face masks lies in agreement with previous research into the effects of occlusion of the lower part of the face in several emotional expressions (e.g.^18–21^). This especially holds for the identification of happy expressions, for which people rely more on the mouth-region; in contrast, for identifying angry expressions, the eye-region seems to be the most prominent diagnostic cue^21–26^. However, most of the studies on the effects of facial masks used facial stimuli with full prototypical emotional expressions (e.g., happy, angry, surprise, fear, sadness, disgust), ignoring the fact that emotional expressions during our daily life are often less intense and not profoundly demarcated. As such, facial expressions are often ambiguous, making their interpretation more susceptible to a perceptual (stimulus driven) or preconceptual (top-down) bias^21,27–34^. Given this ambiguity of emotional expressions in daily life, the question arises how facial masks affect the interpretation of facial expressions. For example, the occlusion of a moderately friendly smile might result in an interpretation of the expression based on the eyes only, which might especially be problematic when the smile is not completely sincere and only used as a social gesture or even used to mask negative feelings^35,36^. In this sense occlusion of the mouth results in a loss of sensory information which may result in a *perceptual (stimulus-driven) bias* away from smile-driven friendliness^37,38^.

On the other hand, as already introduced above, masks may also add (unintended) perceptual information. For instance, although wearing a facial mask during the Covid-19 pandemic is mostly accepted^9^, facial masks can still elicit a negative association due to occlusion of important parts of the face^9,39–43^, which might in turn result in a tendency to interpret an ambiguous emotional expression as a negative, unfriendly appearance. Such contextual (goal-directed) effects have proven to affect the interpretation of emotional expression as well, resulting in a *preconceptual (top-down) bias*^27,28,38^.

In sum, both the perceptual (stimulus-driven) and the preconceptual (top-down) perspectives, predict that masks will elicit a stronger tendency (bias) to classify facial expressions more often as negative (e.g., unfriendly). To investigate whether facial masks elicit such perceptual and/or preconceptual biases in the interpretation of ambiguous emotional expressions, we conducted an experiment in which participants were asked whether ambiguous happy or angry expressions, with and without facial masks, are perceived as friendly or unfriendly.

**Figure 1:**
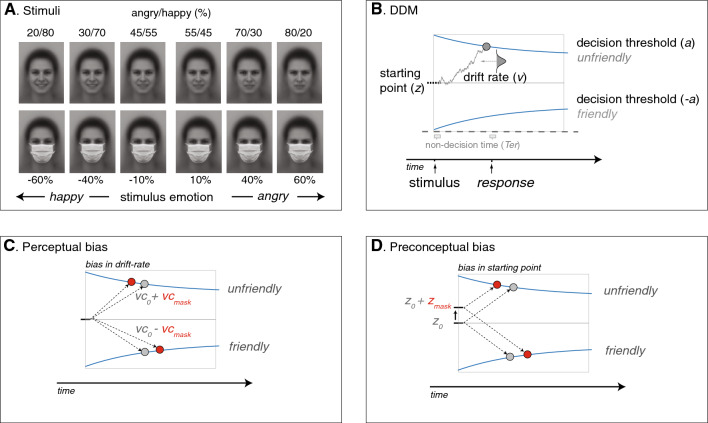
Ambiguous facial stimuli and model predictions. (**A**) Average non-binary happy and angry faces were created by morphing the (average) male and female AKDEF^62^ faces. Next, emotionally ambiguous faces were created by morphing the happy face towards the angry face in 41 incremental steps of 2.5% each. Six faces were used, each with a different angry/happy ratio. Stimulus emotion was defined by the difference in the terms of the ratio (Angry—Happy). (**B**) The DDM (Drift Diffusion Model) represents decisions as an accumulation of noisy sensory evidence over time (drift rate *v*), which starts at starting point (*z*) and ends at one of the decision thresholds (*a or -a*). These decision thresholds are collapsing, meaning that they get closer together as the deadline for making a decision approaches, allowing to model the increasing sense of urgency that individuals experience as they approach a decision deadline. Non-decision time (*Ter)* is the time for processes other than the decision process, such as sensory encoding and execution of the response (grey boxes). (**C**) Perceptual bias is driven by a shift in the drift-rate criterion (*vc*), which determines the point at which the perceptual evidence of the facial expression is classified as either unfriendly or friendly. Due to the ‘*unfriendliness’* of the mask, the drift-rate criterion will shift in favor of the unfriendly alternative, resulting in a biased accumulation process towards the unfriendly (*v*_*0*_ + *v*_*mask*_) and away from the friendly alternative (*v*_0_ −* v*_*mask*_). (**D**) Preconceptual bias is driven by a shift in starting point (*z*), reflecting asymmetric distances to the decision thresholds. Due to an initial *unfriendly* preconception about the mask, the participant expects that the unfriendly alternative will be the correct one, resulting in a starting-point closer to the unfriendly decision threshold (*z*_*0*_ + *z*_*mask*_). In contrast to a shift in the drift-rate criterion, where the stimulus is evaluated differently (1C), a shift in the starting-point does not affect the evaluation process, but rather affects how much evidence is needed for each response. Gray and red dots represent unbiased and biased responses respectively.

Perceptual and preconceptual biases are hard to separate, as both biases result in faster and more choices for a favored alternative. To distinguish between a possible perceptual or a preconceptual bias, we use the drift diffusion model (DDM)^44–46^. The drift–diffusion model allows to decompose the underlying choice process and quantify a possible perceptual or preconceptual bias by utilizing both accuracy and reaction time data^46–48^. The model has been successful applied to distinguish between preconceptual and perceptual biases in various social and motivational decision-making paradigms (e.g.^46,49–52^) as well as in studies investigating biases in fundamental perceptual processes (e.g.^47,53–57^). The DDM assumes that, during a perceptual choice, noisy sensory evidence accumulates until a decision threshold is hit (Fig. 1B; for reviews see refs.^45,48,58–61^). For instance, when a facial mask affects the uptake of *friendly* information (e.g., smile) at the stimulus level, the accumulation process will change in favor for the unfriendly alternative, resulting in a perceptual bias, with faster and more ‘unfriendly’ choices (see Fig. 1C). This process is thus sensitive to *stimulus-driven* biases, but at the same time the starting-point of evidence accumulation can differ based on *top-down* priors. For example, a preconception towards the *unfriendly* choice may lead to a lower decision threshold for the unfriendly option, positioning it closer to the starting point. This asymmetric positioning between the two decision thresholds is equivalent to an asymmetric positioning of the starting point and results in a choice bias, generating more and faster responses for the unfriendly alternative as less evidence is required to meet the *unfriendly* decision threshold (see Fig. 1D).

Even when these biases result in similar behavioral changes, drift–diffusion modelling will allow us to disentangle them and answer the question whether the face-mask driven impairments in the interpretation of ambiguous facial expressions are due to perceptual and/or preconceptual biases.

To test for a possible perceptual or preconceptual bias, we fitted the DDM to participants’ performance on judging emotionally ambiguous faces, with and without mask, on friendliness. Overall, we expect lower drift-rates for masked facial expressions, reflecting less sensory information to make the correct decision, resulting in slower and more error prone choices. In addition, our methodology allows to disentangle a perceptual from a preconceptual bias in the assessment of the friendliness of masked and unmasked ambiguous facial expressions. We expect that if such a preconceptual bias is indeed present when assessing masked faces, the distance between the start and end point of the accumulation process will be smaller for the unfriendly compared to friendly alternative, due to the negative connotations typically associated with face masks^40,42,43^. Furthermore, we will test whether facial masks induce a perceptual (stimulus-driven) bias due to the occlusion of the mouth region and possibly due to the diagnostic cues in the visual features of the mask itself.

## Results

Below we will first report the effects of stimulus emotion and mask on choice and response time data. Next, we will disentangle perceptual and preconceptual biases using DDM analyses that explain the descriptive results in terms of parameter changes.

### Descriptive results

**Figure 2:**
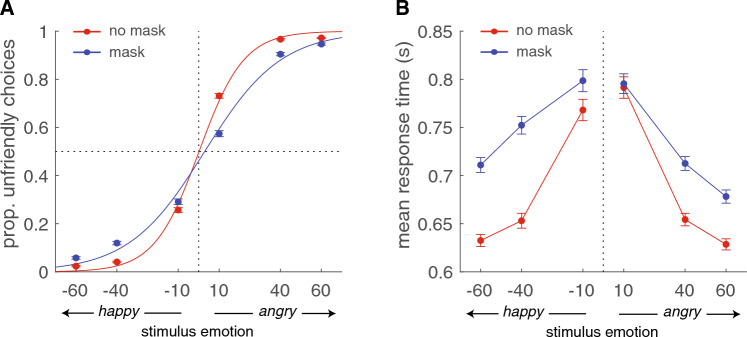
Descriptive data. (**A**) Psychometric functions of the pooled data across participants for masked (blue) and unmasked (red) ambiguous facial expressions. The proportion of unfriendly choices is plotted as a function of stimulus emotion. (**B**) Group averages of median response times (in seconds) as a function of stimulus emotion, for masked (blue) and unmasked (red) ambiguous facial expressions.

To quantify the effect of facial mask on the interpretation of ambiguous facial expressions, a logistic function was fit on the choice data (Eq. 1; Fig. 2A). For both masked and unmasked ambiguous facial expressions, the proportion unfriendly choices increased as a function of stimulus emotion (from happy to angry: see Fig. 2A). For masked faces, there was a small, but significant negative choice bias (β0) reflecting a tendency to choose for friendly more often (b0_mdn_ = − 0.22, one-sample Wilcoxon signed-rank test for b0_mdn_ = 0, V = 2793, *P* < 0.01). No significant bias was found for unmasked faces. Sensitivity (b1) to the stimulus was significantly lower for masked (b1_mdn_ = 6.47) vs unmasked (b1_mdn_ = 10.91) facial expressions (Wilcoxon signed-rank test, W = 9151, *P* < 0.01).

We tested for significant effects in response times using a 2 (masked vs. unmasked) × 2 (happy vs. angry) × 3 (stimulus emotion) repeated measures analysis of variance (ANOVA). For response times, there was a significant main effect of emotional ambiguity of the facial expression, with increasing response times for lower ambiguity levels (stimulus emotion − 10 and 10), symmetrical around zero intensity, *F*(1,135) = 366.4, *P* < 0.01. The main effect of mask was significant as well, with slower response times for masked stimuli, *F*(5,675) = 290.5, *P* < 0.01. In addition, there was a significant interaction effect between the stimulus emotion of the facial expression and mask, indicating that the effect of mask was not equally distributed across ambiguity levels, *F*(5,675) = 44.1, *P* < 0.01. More specifically, the difference between response times for masked and no-masked facial expression became smaller for the high (− 10, 10) ambiguity levels (see Fig. 2B). Furthermore, post-hoc t-test show significant slower response times for masked happy than for masked angry facial expressions with a low (− 60 vs 60) or moderate (− 40 vs 40) ambiguity in stimulus emotion (both *ts*(135) > 5.4, *P* < 0.01). No such difference was found for the facial stimuli without a mask. Instead, participants were slower for angry vs happy facial expressions without a mask, with high emotional ambiguity (10, vs − 10), *t*(135) = 3.82, *P* < 0.01.

Overall, these results of the analyses of choice and response times show that there are small, asymmetrical effects of a facial mask on interpretation of ambiguous emotional expressions. To further quantify these effects, we fitted the DDM to the data allowing us to decompose the effects in the underlying choice parameters.

### DDM analyses

The RT results in Fig. 2 partly suggest a bias towards unfriendly choices for masked stimuli, showing faster choices for easy (60) and moderate (40) angry masked faces. In contrast, the psychometric data reflect a general loss of sensitivity combined with an unexpected bias to friendly choices in the mask condition. These contradictory findings suggest that facial masks might affect the interpretation of ambiguous emotional faces via different underlying mechanisms. To identify whether bias effects are driven by a preconceptual (top-down) or perceptual (stimulus-driven) process, the diffusion model was fitted to both the RT and choice data simultaneously, allowing to disentangle these different types of bias.

**Figure 3:**
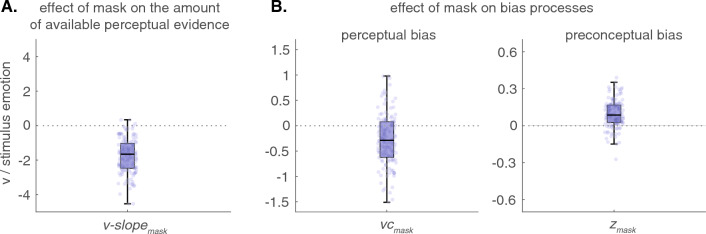
Effects of facial masks on the amount of available perceptual information (*v-slope*) and bias parameters (*vc* and *z*). (**A**) The presence of facial masks affects the relationship between the stimulus emotion and the drift-rate, resulting in a less pronounced increase in evidence accumulation with each increase in stimulus emotion (*v-slope*_*mask*_). (**B**) Effect of facial masks on drift-rate criterion (*left*: *vc*_mask_) and starting point (*right*: *z*_*mask*_) relative to unmasked facial expressions. Values > 0 indicate a bias towards the unfriendly alternative, while values < 0 indicate a bias towards the friendly alternative.

**Figure 4:**
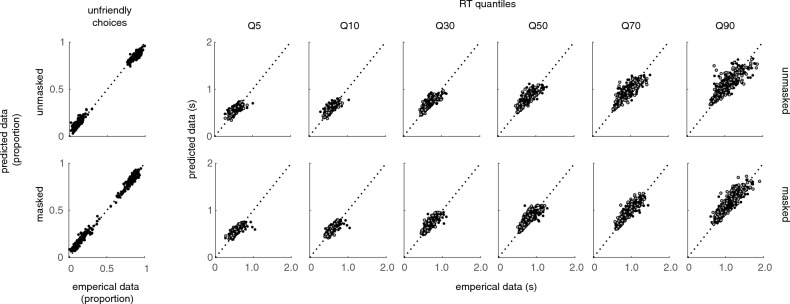
Goodness of fit of the *full*_*vc,z*_ model. The graph shows empirical (x-axis) and predicted (y-axis) data for unmasked (top row) and masked (bottom row) facial stimuli. Predicted data was generated for each participant separately using the individual *full*_*vc,z*_ model parameters. Choice data was plotted together with the quantiles (5th, 10th, 30th, 50th, 70th, 90th) of the RT distributions using RTs for unfriendly (black) and friendly (white) responses on happy and angry facial expressions (collapsed over intensity levels). The plot shows that the predicted values of choice data and RT quantiles are close to the empirical values for most datapoints, suggesting that the model fit the data reasonably well.

For the diffusion-model fits (see the methods section for model selection and Fig. 4 for goodness-of-fit) we found that facial masks lead to a reduced slope for the increasing emotional change (mean(SD) *v-slope*_*mask*_ = − 1.78(1.1), Wilcoxon signed-rank test, *V* > 9294, *P* < 0.001; see Table 1 and Fig. 3A), relative to the slope of the unmasked facial expressions (mean(SD) *v*_*0*_ = 5.68(0.11) ). This indicates that drift-rate increases less steeply for the emotional change of masked facial expressions compared to unmasked ones.

**Table 1:** Mean(SD) parameter values for the full_vc,z_ model.

	*a*	*tau*	*Ter*	*vc*	*v-slope*	*z*
Unmasked _*0*_	1.08 (0.23)	1.29 (0.5)	380 (61)	0.17 (0.36)	5.68 (0.11)	− 0.06 (0.08)
Effect of mask _*mask*_			14 (26)	− 0.28 (0.53)	− 1.78 (1.10)	0.10 (0.11)

All parameter values were significantly different from zero (one-sample Wilcoxon signed-rank test, all Vs > 1561, ps < 0.01); a = decision threshold; tau = collapsing rate; Ter = non-decision time; vc = bias in drift-rate (drift-rate criterion); v-slope = slope of the linear relationship between drift-rate and stimulus emotion; z = starting-point.

To test for a perceptual bias, we fitted an additional parameter (*vc*_*mask*_) to capture a possible change in the drift-rate criterion (vc_0_) due to the facial masks. We found a significant negative effect of mask, showing a perceptual bias towards the friendly alternative (mean(SD) *vc*_*mask*_ = − 0.28(0.53), Wilcoxon signed-rank test, *V* > 2107, *P* < 0.001; see Table 1 and Fig. 3B) for masked facial expressions, relative to unmasked facial expression with mean(SD) *vc*_*0*_ = 0.17(0.36).

In addition to a perceptual bias, we tested for a preconceptual bias by adding an additional parameter (*z*_*mask*_) to the model that captures possible shifts in the starting-point (*z*_*0*_). We found that masks increased the starting-point of the decision process with mean(SD) *z*_*mask*_ = 0.10(0.11) (Wilcoxon signed-rank test, *V* > 8331, *P* < 0.001) relative to the unmasked condition (*z*_*0*_ = − 0.06(0.08)). This additional shift in the starting-point of the accumulation process indicates a significant preconceptual bias towards the unfriendly alternative for masked facial expressions (see Table 1 and Fig. 3B).

In addition to drift-rate slope (*v*), starting point (*z*) and drift-rate criterion (*vc*), we tested whether masks affected the early sensory processes prior to the accumulation process, represented by non-decision time (*Ter; see *Fig. 1). Facial masks increased non-decision time with mean(SD) *Ter*_*mask*_ = 14(26) ms relative to the unmasked facial expressions (mean(SD) *Ter*_*0*_ = 380(61) ms), resulting in slower non-decision times for masked facial expressions.

In sum, our descriptive analyses suggest that masked compared to unmasked faces are judged as more friendly, but that judging masked friendly faces takes more time. Our DDM analyses show that masking a face results in a loss of sensory information and in an unfriendly preconception towards facial expressions. In contrast, we found a friendly perceptual bias as well. This suggests that, although diagnostic cues in masked faces bias our participants towards friendliness via a stimulus-driven process, our participants also have the preconception that masked faces are unfriendly.

## Discussion

To investigate whether face masks induce a perceptual or preconceptual bias in the interpretation of ambiguous facial expressions, participants performed a task in which they had to decide whether ambiguous facial expressions, with or without a mask, were perceived as friendly or unfriendly. We fitted a drift–diffusion model (DDM) to their performance data to test for the independent effects of perceptual and preconceptual biases in these decisions. As expected, the analyses of descriptive data showed a lower sensitivity for the masked emotional expressions, generally resulting in slower and less correct responses for the masked compared to the unmasked facial expressions. This was also supported by our Drift Diffusion Model (DDM) analysis. Here, we found that mask had a decreasing effect on the strength of the relationship between stimulus emotion and drift rate for masked faces (*v-slope*_*mask*_), suggesting that less information was available during the decision process. These effects are in line with studies showing that covering the mouth decreases the amount of available information to correctly recognize and identify an emotional expression^7–14,14–16,18–21^.

In addition, quantification of choice data using the psychometric function shows a small but significant bias toward *friendly* faces for masked but not for unmasked facial expressions. This is unexpected, since the mouth is often considered to be more important in the recognition of happy facial expressions compared to the recognition of angry facial expressions^21–26^. As such, based on perceptual processes alone, we expected that wearing a facial mask would especially hamper the identification of happy facial expressions, resulting in a bias *away* from the friendly choices for masked stimuli. Instead, the small perceptual bias towards friendly choices suggests that covering the mouth with a facial mask has a larger effect on the misinterpretation of angry than happy facial expressions, which seems to be particularly the case for expressions with a high emotional ambiguity (i.e., − 10 and 10; see Fig. 2A). In contrast, analyses of average response times show slower response times for happy than for angry masked facial expressions with low (− 60 vs 60) or moderate (− 40 vs 40) emotional ambiguity. These contradictory findings in the descriptive data underscore the importance to fit a computational model to the data that considers both choice and response time data, allowing us to disentangle the underlying biasing mechanisms.

To measure possible systematic perceptual (stimulus driven) or preconceptual (top-down) biases in the interpretation of masked and unmasked facial expressions, we fitted the drift–diffusion model to each participant's choice and response time data. Results show that facial masks affect both the perceptual and preconceptual processes, in opposite direction, with a preconceptual bias towards unfriendly and a perceptual bias towards friendly choices.

As expected, we found a small but significant shift in the starting point towards the unfriendly alternative for masked faces, relative to the unmasked condition. This bias in starting point suggests that participants start the decision process with asymmetrical decision thresholds (i.e., smaller for unfriendly vs friendly, in masked choices), resulting in faster and more choices for the unfriendly alternative. Such a lower threshold might indicate a top-down preconception in which the alternatives already have a different representation for masked versus unmasked stimuli, prior to the initial choice. This might be due to the somewhat threatening connotation of the mask, providing a context which might bias the interpretation of ambiguous facial expressions^27,28^.

In addition to the preconceptual (top-down) bias observed for masked faces, we identified a perceptual (stimulus-driven) bias favoring *friendly* sensory information for masked faces, relative to the unmasked condition. One explanation for this unexpected perceptual bias may be related to diagnostic features of the mask itself. Studies investigating the impact of the emotional intensity of the facial expression show that happy expressions are more easily detected, even at low intensities^63^ and resolutions^64^. Given the difference in the detectability of happy vs angry at a low emotional intensity, it might be the case that the low-level visual features of the mask are closer to a happy than an angry mouth expression. This might particularly be the case for early perceptual processes that are primarily affected by low-level visual features^38,65,66^ in which the mask-features might result in a surrogate smile for the face, biasing the effect away from unfriendliness. As such, the drift-rates might be biased towards sensory evidence in favor for the *friendly* alternative for masked faces, due to the asymmetry in happy/angry sensitivity, where the effect of *happy-information* in the mask itself is stronger than the angry diagnostic features in the eyes of masked faces. Furthermore, it has been shown that low spatial frequencies of facial expressions are faster and earlier processed in the information stream, compared to high spatial frequencies^38,65,67,68^. In light of this explanation, our choices might be biased first by the outstanding low-level spatial features (stimulus-driven), while the semantic (top-down) categorization based on the (asymmetric) decision thresholds is processed later in time^65,66^. Conceptually, the classic view of the DDM states that starting point effects are determined prior to the decision process. However, it is possible that the bias resulting from the asymmetric decision thresholds becomes more prominent in a later stage of the decision process.

Note that, in addition to the biasing effects of facial masks, both the baseline starting point (*z*_*0*_) and drift-rate criterion (*vc*_*0*_) demonstrated a slight but significant shift opposite to the effects of the masked condition (see Table 1). The negative initial starting point values could imply that our participants have the preconception that emotionally ambiguous faces are friendly, but this positive bias could also reflect a reversed effect on faces when the mask is *not* visible, resulting in a more positive connotation than usual. On the other hand, the positive drift-rate criterion towards *unfriendly* choices for unmasked faces might suggest that emotionality is not symmetrically distributed across the morphed dimension between happy and angry facial stimuli. This asymmetry seems in turn to disappear after adding a mask to the faces, suggesting that this asymmetry is driven by the mouth region. Future research that includes a condition with a mouth that is covered, but not by a mask, can resolve this issue by showing whether the perceptual bias towards friendliness is due to the additive effect of the mask, or due to the reduction of asymmetry in ambiguity by covering the mouth.

Several limitations must be noted in interpreting our findings. One limitation is the inconsistency observed in the model selection process. While both AIC and BIC were used for model-selection, their values point to different winning models. The *full*_*vc,z*_ model with both a variable starting point (*z*) and drift-rate criterion (*vc*), for instance, yielded the lowest average AIC. Furthermore, this model was the most suitable one for the largest fraction of participants (32%). Conversely, the average BIC was lowest for the model that only had the variable drift-rate criterion (*vc*), implying a better fit by this metric. Despite the lower average BIC values for the *vc*-only (*reduced*_*vc*_) model, it is noteworthy that for more than half of the participants (54%) the *null* model fitted their data at best. This discrepancy in criteria suggests a certain degree of uncertainty in the model selection and calls for careful interpretation of the models' outcomes. Given the substantial variability in model selection among participants, a reasonable argument could be made for fitting the *full*_*vc,z*_ model as it would capture all effects, including null effects, thus also accounting for participants who did not exhibit any biasing results. However, it's important to acknowledge that the effects of the facial masks on the starting point were relatively small, which could potentially be attributed to overfitting of the model. Although our model comparison approach is aimed to address this possible issue, the divergence in AIC and BIC results highlights the need for further investigation.

In sum, we investigated whether face masks induce a loss of information and perceptual or preconceptual biases, participants were asked to decide whether masked or unmasked ambiguous facial expressions were perceived as friendly or unfriendly. Results show that wearing a face mask causes a loss in sensory information and a preconceptual bias towards *unfriendly* but a perceptual (stimulus-driven) bias towards *friendly* choices for masked faces. These results suggest that people have a prior top-down tendency to interpret facial masks as unfriendly, regardless of the friendly (stimulus-driven) effects of the facial mask itself.

## Methods

### Participants

Participants (n = 145, mean(std) age = 22.3(4.4), 109 female) were invited via online media or Utrecht University’s Sona Systems (https://www.sona-systems.com/) to participate in an online experiment in exchange for course credit. Nine participants were excluded based on insufficient performance on the task (see descriptive analyses below). Informed consent was obtained from all participants. The experiment was approved by and was in accordance with the guidelines and ethical standards of the the Ethics Committee of Utrecht University (EC-FETC18-129).

### Materials and Stimuli

*Face stimuli* were adapted from the Averaged Karolinska Directed Emotional Faces (AKDEF^62^). First, to control for possible sex differences in facial expressions^69,70^, we created an angry and a happy *non-binary* face by morphing the average (resp. angry and happy) male and female faces to each other using WinMorph (version 3.01). Next, emotionally ambiguous faces were created by morphing the happy face towards the angry face in 41 incremental steps of 2.5% each. From this range of morphed non-binary facial stimuli with different angry/happy ratios, eight ambiguous expressions were chosen. For each face, a masked version was created, by adding surgical mask to each face using Adobe Photoshop (version 22.2). A normal surgical mask was chosen, as these were seen commonly in public at the time of data collection. The color of the masks was adjusted to resemble the black-and-white coloring of the images of the faces. The edges of the mask were softened to incorporate it more naturally into the image.

Six of the facial expressions with ratios (angry/happy%) of 80/20%, 70/30%, 55/45%, 45/55%, 30/70% and 20/80%, with and without a mask, were used as main stimuli (see Fig. 1B). Two facial expressions (60/40% and 40/60%), with and without a mask served as filler trials to add more variance to the stimuli, reducing predictability of the six main facial stimuli. Stimulus emotion was expressed as the difference between the percentage happy and angry facial expressions (assuming 50/50 to have 0% evidence for either a happy or angry expression and thus full ambiguity) resulting in 6 (signed) emotion levels of − 60%, − 40%, − 10% for happy and 10%, 40% and 60% for angry facial expressions.

### Procedure

A two-alternative forced (2AFC) choice task was set up and hosted on Gorilla Experiment Builder (www.gorilla.sc)^71^. After consent was given, general demographic information was collected after which the participant was assigned to one of the two (counterbalanced) versions of the 2AFC-task. In the 2AFC-task, participants were asked to respond as quickly as possible to decide whether the facial expression was perceived as friendly or unfriendly, for a total of 608 trials. These trials consist of 96 trials (48 masked) for each of the six stimulus emotions (− 60%, − 40%, − 10%, 10%, 40%, 60%) and 32 (16 masked) filler-trials (stimulus emotions − 20% and 20%). To keep the participants engaged, the experiment was divided into 8 blocks of 76 trials each. Each block contained a random alternation of all possible conditions (mask x stimulus emotion).

Each trial started with a fixation cross that was presented for a randomly chosen duration between 600 and 1200 ms to prevent anticipatory responses to the stimulus. Next, the stimulus was shown on the screen, during which the participant was required to respond with the ‘C’- or ‘M’-key to indicate their choice. Stimulus display was terminated after a button press or a time-out of 2300 ms. Choice associations with these responses (‘friendly’ or ‘unfriendly’) were counterbalanced between participants. We chose to use the labels *friendly* and *unfriendly* since the created images were not fully ‘angry’ or ‘happy’. For example, the ambiguous facial expression with 10% stimulus emotion might not be perceived as angry perse, but still has a mild ‘unfriendly’ expression. Subsequently, the participant’s response feedback was shown for 400 ms (a green check for correct and a red cross for false responses). Whenever a response was made throughout the fixation cross period, an icon with the words “too fast” appeared. If subjects did not make a response within the given response time (2300 ms), the word “miss” was shown. Missed trials were excluded from analyses as response times of > 2300 ms fell well beyond the upper bound of the interquartile range of RTs across the group (1228 ms).

### Analyses

#### Descriptive data

For each participant, response times were log transformed, after which for each condition response times were removed that were three standard deviations away from the average response time (on average, 4.7% of the data). Next, median response times were calculated for each condition separately. A 2 (masked vs. unmasked) × 2 (happy vs. angry) × 3 (stimulus emotion) repeated measures ANOVA was used to test for effects of mask, choice and emotion on response times.

To quantify effects of mask and stimulus emotion on choice performance, a logistic function (see Eq. 1) was fit onto the choice data for each participant.

1 $$ p_{unfriendly} = \frac{1}{{1 + e^{{ - \left( {\beta_{0} + \beta_{1} S} \right)}} }} $$

This function included two terms, an emotion-dependent term that reflected sensitivity to the stimulus and an emotion-independent term that reflected a choice bias towards either ‘friendly’ or ‘unfriendly’ choices. Non-parametric Wilcoxon t-tests were used to test for a difference in sensitivity (*b*_*1*_) between masked and unmasked faces and for a possible choice bias (*b*_*0*_) in the masked and unmasked conditions. Based on these initial analyses, nine participants were excluded based on a significantly large deviance between empirical data and the fit of the psychometric function (all nine deviances > 310; *X*^2^(df's < 280) < 242.2, *P* = 0.05).

#### DDM analysis

In order to examine a possible perceptual or preconceptual bias in the behavioral data, we fitted the drift–diffusion model (DDM^44^; see Fig. 1B) to each participant's choice and response time data simultaneously using the pyDDM Python package^72^. Our aim was to determine which model parameters could account for a potential choice bias for masked facial expressions. Consequently, we performed model selection using four models to each participant's data: (1) a null model, (2) a reduced_*vc*_ model including a perceptual bias (Fig. 1C), (3) a reduced_*z*_ model incorporating a preconceptual bias (Fig. 1D), and (4) a full_*vc,z*_ model that integrates both preconceptual and perceptual biases (see also^49–51,53,55–57^). Models were fitted to the data employing maximum likelihood estimation. Furthermore, we calculated the Akaike Information Criterion (AIC) and the more conservative Bayesian Information Criterion (BIC) to select the model exhibiting the best goodness-of-fit, as indicated by the lowest AIC or BIC value.

For the null model, we assumed a linear relationship between drift-rate and stimulus emotion *k* (with values − 60, − 40, − 10, 10, 40, 60). The drift-rate incorporated an intercept (*vc*_*0*_), a slope (*v-slope*_*0*_), and an additional term (*v-slope*_*mask*_) to capture the effect of facial masks. Consequently, the drift-rate was defined as follows:

2 $$ \upsilon = \upsilon c_{0} + k \cdot \left( {\upsilon - slope_{0} + C \cdot \upsilon - slope_{mask} } \right) $$

where *C* = 1 for masked and *C* = 0 for unmasked facial expressions. To capture possible changes in early sensory and late motor processes, we fitted non-decision time effect *Ter*_*0*_ with an additional term *Ter*_*mask*_ for masked faces. Non-decision time *Ter* was defined as:

3 $$ Ter = Ter_{0} + C \cdot Ter_{mask} $$

where *C* = 1 for masked and *C* = 0 for unmasked facial expressions.

In addition to drift and non-decision time parameters, we included a starting point *z*_*0*_ and an exponentially collapsing decision threshold (*a*, with *tau* reflecting the rate of collapse). The collapsing decision threshold was added to the model to account for urgency effects due to the decision deadline^55–57,57,73–75^. Both *z*_*0*_ and *a* were fixed across the masked and unmasked conditions. Variability parameters (*sz*, *st0* and *sv*) were not fitted and set to zero as fitting these parameters can bias the estimations of the main parameters^76–78^. However, since variability in starting-point (*sz*) can account for fast-errors, which in turn can explain the (opposite) effects of the mask on choice and RT data, we repeated our model-selection procedure with *sz* added to each model (see Supplementary Material). Adding *sz* to the four models did not change the outcome of our model-selection procedure. Neither did it change the outcome of starting-point differences (see Table S1).

The *reduced*_*vc*_ model, which incorporates perceptual bias, extends the null model by including an additional drift-rate term, *vc*_*mask*_ in Eq. 2 to account for a possible bias on the drift-rate criterion (intercept *vc*_*0*_). In this case, drift rate (v) is defined as follows:

4 $$ \upsilon = \upsilon c_{0} + C \cdot \upsilon c_{mask} + k \cdot \left( {\upsilon - slope_{0} + C \cdot \upsilon - slope_{mask} } \right) $$

where *k* is stimulus emotion and *C* = 1 for masked and *C* = 0 for unmasked facial expressions.

The *reduced*_*z*_ model, which incorporates preconceptual bias, extends the null model by including an additional starting-point term *z*_*mask*_ to account for possible bias on the starting point of the accumulation process, relative to the unmasked condition (*z*_*0*_). As such, starting point was defined as:

5 $$ z = z_{0} + C \cdot z_{mask} $$

where *C* = 1 for masked and *C* = 0 for unmasked facial expressions.

Finally, the *full*_*vc,z*_ model included all parameters of the *null* model with both additional terms *vc*_*mask*_ and *z*_*mask*_ to account for a possible perceptual and preconceptual bias respectively.

Model selection shows that the full_vc,z_ model outperforms the null and reduced_vc and z_ models, which is reflected in both the average AIC values and the proportion of participants for whom this model was the best fit (see Table 2). For BIC values, the picture is less distinct: Here, average BIC values are lowest for the reduced_vc_ perceptual bias, but with a slight difference compared to the reduced_z_ preconceptual bias model (difference reduced_vc – z_ = 0.21). Notably, for a large faction of participants (54%) the null model seems to be the best model describing their behavioral data. Given the substantial variability in model selection among participants and the better fit of the full_vc,z_ model according to the less conservative AIC, we decided to continue our analyses using the full_vc,z_ model to capture the individual differences in parameter values (see Fig. 4 for a visual representation of the Goodness of fit).

**Table 2:** Average criteria values and the percentage of participants (n%) for which the model had the lowest criteria.

	*Null*	*reduced*_*vc*_	*reduced*_*z*_	*Full*_*vc,z*_
Mean AIC (n%)	− 166.34 (21%)	− 172.21 (24%)	− 172.42 (23%)	− **174.55** (32%)
Mean BIC (n%)	− 131.88 (54%)	− **133.66** (18%)	− 133.45 (22%)	− 131.49 (5%)

Lower criteria values indicate a better fit.

*AIC* Akaike information criterium, *BIC* Bayesian information criterium.

Lowest values are in bold.

Finally, a one sample Wilcoxon signed-rank tests was used to test whether the additional bias effects *z*_*mask*_* and vc*_*mask*_ were significantly different from zero.

### Supplementary Information

Supplementary Information.

## Data Availability

The datasets used and/or analysed during the current study are available from the corresponding author on reasonable request.
