# Supplementary material for: Drift–diffusion modeling reveals that masked faces are preconceived as unfriendly
Source: Sci Rep. 2023 Oct 9;13:16982. doi: 10.1038/s41598-023-44162-y (PMC10562405; doi:10.1038/s41598-023-44162-y)
Supplement: Supplementary file 1 — Supplementary Information. [file 41598_2023_44162_MOESM1_ESM.docx]

**Supplementary material**

**Drift-Diffusion Modeling Reveals that Masked Faces are Preconceived as Unfriendly**

*Martijn J. Mulder^1^, Franziska Prummer^2^, David Terburg^1^, J. Leon Kenemans^1^

^1^ Department of Experimental Psychology, Helmholtz Institute, Utrecht University, The Netherlands

^2^ School of Computing and Communications, Lancaster University, United Kingdom

The behavioral results in the main manuscript show that for the masked condition, there is a choice bias in favor of friendly but an RT bias in favor of unfriendly choices. Although studies have shown that estimations of variability parameters can have an impact on estimations of the main parameters (Boehm, U. *et al, 2018;* Lerche, V. & Voss, 2017; Tillman, Van Zandt & Logan, 2020), behavioral results might suggest that across-trial variability in starting point (*sz*) is needed to account for fast-errors underlying the data, which might have an impact on the starting-point estimations (Laming, 1968; Ratcliff & Rouder, 2000; Ratcliff & McKoon, 2008).

To test for possible *sz* effects, we re-ran our model-selection procedure in pyDDM (Shinn et al.,2020), while adding *sz* to our models. As shown by Table S1, adding *sz* does improve the AIC values for all models, compared to the models without *sz* as shown in Table 1 (see manuscript). However, adding *sz* does not add to the distinctiveness of our model-selection procedure. That is, for AIC, the *Full_vc,z+sz_* model still outperforms the other reduced and null models. In contrast, for BIC values, the *sz*-models have somewhat higher (less negative) BIC values compared to the models without *sz*, which is expected since BIC is more conservative and adds a stronger penalty to the number of model parameters.

|  | Table S1 – Average criteria values and the percentage of participants (n%) for  which the model had the lowest criteria. All models included variability in starting-point (*sz*) as an additional parameter. AIC: Akaike information criterium, average BIC: Bayesian information criterium | | | |
| --- | --- | --- | --- | --- |
|  | *Null_sz_* | *reduced_vc_* | *reduced_z_* | *Full_vc,z_* |
| mean AIC (n%) | -168.18 (24%) | -174.46 (21%) | -173.99 (23%) | **-176.46** (32%) |
| mean BIC (n%) | -129.42 (55%) | **-131.40 (**19%) | -130.92 (21%) | -128.91 (5%) |
|  | *(note: lower criteria values indicate a better fit)* | | | |

In addition to the comparisons between AIC and BIC criteria, we also tested the extent to which *sz* changed our starting-point values of interest. Although starting point values are somewhat smaller for the *Full_vc,z+sz_* model when *sz* is included (*z_mask_sz_* = 0.088(0.10) vs *z_mask_* = 0.095(0.11)) results are significant and do not change the main message of our manuscript.

**References**

Ratcliff, R., & Rouder, J. N. (2000). A diffusion model account of masking in two-choice letter identification. Journal of Experimental Psychology: Human perception and performance, 26(1), 127.

Laming, D.R.J. (1968). Information theory of choice-reaction times. Academic Press.

Ratcliff, R. & McKoon, G. The diffusion decision model: theory and data for two-choice decision tasks. *Neural Comput.* **20**, 873–922 (2008).

Boehm, U. *et al.* Estimating across-trial variability parameters of the Diffusion Decision Model: Expert advice and recommendations. *Journal of Mathematical Psychology* **87**, 46–75 (2018).

Lerche, V. & Voss, A. Retest reliability of the parameters of the Ratcliff diffusion model. *Psychological Research* **81**, 629–652 (2017).

Tillman, G., Van Zandt, T. & Logan, G.D. Sequential sampling models without random between-trial variability: the racing diffusion model of speeded decision making. *Psychon Bull Rev* **27**, 911–936 (2020). https://doi.org/10.3758/s13423-020-01719-6

Shinn, M., Lam, N. H., & Murray, J. D. (2020). A flexible framework for simulating and fitting generalized drift-diffusion models. *ELife*, *9*, e56938. https://doi.org/10.7554/eLife.56938
